# Mass kills in hatchery-reared European seabass (*Dicentrarchus labrax*) triggered by concomitant infections of *Amyloodinium ocellatum* and *Vibrio alginolyticus*

**DOI:** 10.1080/23144599.2022.2070346

**Published:** 2022-05-04

**Authors:** Reham H. Ragab, Mamdouh Y. Elgendy, Nader M Sabry, Mahmoud S. Sharaf, Marwa M. Attia, Reda M.S. Korany, Mohamed Abdelsalam, Ahmed S. Eltahan, Elsayed A. Eldessouki, Ghada O. El-Demerdash, Riad H. Khalil, Abeer E. Mahmoud, Alaa Eldin Eissa

**Affiliations:** a Department of Aquatic Animal Medicine and Management, Faculty of Veterinary Medicine, Cairo University, Giza, Egypt; b Department of Hydrobiology, Veterinary Research Institute, National Research Centre, Giza, Egypt; c Fish Disease Lab, Aquaculture Division, National Institute of Oceanography and Fishery (NIOF), Alexandria, Egypt; d Department of Parasitology, Faculty of Veterinary Medicine, Cairo University, Giza, Egypt; e Department of Pathology, Faculty of Veterinary Medicine, Cairo University, Giza, Egypt; f Department of Pathology, Faculty of Veterinary Medicine, Suez Canal University, Ismailia, Egypt; g Department of Fish Health and Diseases, Faculty of Fish Resources, Suez University, Suez, Egypt; h Agriculture Research Centre, Animal Health Research Institute, Al-Fayoum Provincial Laboratory, Giza, Egypt; i Department of Poultry and Fish Diseases, Faculty of Veterinary Medicine, Alexandria University, Alexandria, Egypt; j Department of Fish Diseases, Animal Health Research Institute, Assiut Provincial Laboratory, Agriculture Research Center, Dokki, Egypt

**Keywords:** *Dicentrarchus labrax*, frys, amyloodiniosis, vibriosis, molecular characterization, treatment

## Abstract

Amyloodiniosis and vibriosis are serious diseases in European seabass (*Dicentrarchus labrax*) hatcheries with noticeable high mortality. This study was conducted on tank-cultured *D. labrax* frys at a private marine hatchery near Mariout Lake (Alexandria, Egypt). Frys showed a high mortality rate (70%), lethargy, darkening, asphyxia, ascites, and velvety skin appearance. Both infectious agents were presumptively identified in all investigated frys. The identities of the two recovered agents were confirmed by molecular assay and phylogenetic analysis. On the tissue level, histopathological examination of skin, splenic, and renal tissue indicated severe alterations due to the direct impacts of both infections. On the cellular level, scanning electron micrographs showed both protozoal and bacterial pathogens on/in gill epithelial cells in solitary and colonial forms. *Vibrio alginolyticus* showed variable results for tested antibiotics, with a higher sensitivity to florfenicol. A successful control strategy was strictly adopted to overcome infections and stop mortalities. Copper sulphate and hydrogen peroxide were efficiently applied to tank water to overcome *A. ocellatum* infections. Further, florfenicol was effectively used to overcome systemic *V. alginolyticus* infections. The efficacy of treatments was confirmed by the absence of infectious agents in randomly collected fish samples. To the best of the authors’ knowledge, this study is one of the earliest Egyptian studies that dealt with the dilemma of mass kills associated with external parasitic/systemic bacterial infections among hatchery-reared European seabass.

## 1. Introduction

Mariculture in Egypt is clustered geographically into four provinces, namely Alexandria (Mariout Lake), Damietta (Deeba Triangle), Ismailia, and Port Said (Sahl El-Tina) [1]. The majority of Egyptian mariculture activities regularly rely on wild seed collection from some Northern coastal lakes and the Mediterranean Sea [2]. Mariout Lake is located in Northern Egypt, Southeastern of Alexandria, one of Egypt’s most densely populated areas [3]. Mariout Lake is a source of marine fish production and a highly productive fishing lake [3].

European seabass (*Dicentrarchus labrax* L.) is one of the most important commercial fish species in the Mediterranean basin [4–6]. Egypt is one of the leading producers of farmed *D. labrax*, with 24,914 MT in 2018, accounting for 11.5% of the total aquaculture production [2]. *D. labrax* can withstand a wide range of water temperatures (8°C–24°C) and salinities (3‰–35‰), rendering it an ideal choice for aquaculture. *D. labrax* aquaculture started late in 1980–1985 due to the expansion of hatcheries and improvement of larval survival [7]. *D. labrax* production systems are diverse, including intensive farming in cages/tanks and semi-intensive fish ponds [8–10].

Diseases affecting farmed *D. labrax* are the central challenges for developing and expanding this critical aquaculture sector. Bad management practices and intensification of aquaculture operations exacerbate infections affecting fish, resulting in a wide range of pathologies. Amyloodiniosis and vibriosis are among the most critical infections affecting *D. labrax*, causing mortalities and huge economic losses [9,11,12].

Amyloodiniosis (velvet disease) is a devastating protozoal disease caused by *Amyloodinium ocellatum*, an obligate ectoparasitic dinoflagellate that infects the gills, skin, and buccal cavity of numerous fish species in brackish and marine environments, including *D. labrax* [11,13]. *A. ocellatum* has three developmental life stages: a trophont that attaches to and feeds on fish tissues, a tomont that multiplies via division on the substrate, and an infective dinospore that swims freely, infecting new hosts [14]. *A. ocellatum* resists a wide range of aquatic environmental variables, including salinity ranging from 1 to 60 ppt [15]. Such amazing environmental tolerance allows the aerosol transmission of *A. ocellatum. A. ocellatum* dinospores could be transmitted via aerosol droplets that cover a distance of at least 4 metres from their origin, infecting new fish. *A . ocellatum* feeding activity causes severe damage and injuries to fish tissues, facilitating numerous bacterial invasions [16].

Vibriosis caused by *Vibrio alginolyticus* is a serious bacterial infection affecting many commercially important fish species, including *D. labrax. V. alginolyticus* is a halophilic bacterium that is ubiquitous in marine aquatic environments and frequently affects cohabitant aquatic species when adverse environmental circumstances arise. The pathogen possesses numerous virulence determinants, including powerful extracellular products that potentiate severe infections in wild and farmed fish [17]. High mortality outbreaks relevant to *V. alginolyticus* have been reported worldwide in many fish species, including grouper, trout, sea bream, and European seabass [9,12,18–20]. Affected fish exhibit typical signs of haemorrhagic septicaemia with widespread haemorrhages on external body surfaces, around the base of the fins, on the head, and in the belly. Outbreaks affecting young fish are commonly associated with anorexia, darkening, and sudden death [21].

The present study aimed to unlock or identify the puzzling causes behind the emergent mass kills of hatchery reared European seabass frys in an Egyptian private marine hatchery via clinical, parasitological, bacteriological, molecular and scanning electron microscopy (SEM) techniques. The immunological reactions induced using quantitative real-time polymerase chain reaction (qRT-PCR). Further, a field trial evaluation of a compound treatment strategy to control coinfections and associated mortalities was performed.

## 2. Materials and Methods

### 2.1. Case history and sampling

The high mortality rates (70%) of 1 g weighed European seabass (*D. labrax*) frys were recorded at a private fish hatchery located in the neighbourhood of Lake Mariout (Alexandria, Egypt) in July 2021. Fish were reared in rectangular fibreglass tanks (30 m^3^ each). Fish were stocked at 1,000 fry/m^3^ and fed powdered commercial feed (46% protein and 17% fat) at a 10% feeding rate with 70% daily water exchange. Temperature, salinity, dissolved oxygen, pH, and unionized ammonia averaged 20°C, 33‰, 3.5 mg/L, 9, and 0.3 mg/L, respectively.

One hundred European seabass frys were collected from the affected tanks during the noticed mortality events. Fish were transferred in well aerated water filled plastic bags with minimum delay to the Aquatic Animal Medicine and Management Laboratory (AAMML), Faculty of Veterinary Medicine, Cairo University (Cairo, Egypt). Further clinical, parasitological, bacteriological, and molecular investigations were performed according to Eissa [22], Eissa et al. [20], and Abdelsalam et al. [23].

### 2.2. Parasitological examination

Fish were euthanized by an overdose of MS-222 (Sigma) and dissected under stereoscopic microscope (SZX16; Olympus, Japan). Scrapings from the skin and gills were taken onto slides, fixed with absolute methanol, air-dried, and stained with Giemsa stain, (Sigma–Aldrich). Stained smears were washed after 20–30 min, left to dry and examined carefully under a light microscope (CX33; Olympus, Japan). According to Sindermann [24], Cruz-Lacierda et al. [11], and Eissa et al. [25], protozoan parasites were identified according to the shape of the trophont parasites with their attachment stalks. All measurements were recorded as mean ± S.D. The prevalence and mean intensity of parasitic infestations were estimated and reported.

### 2.3. Scanning electron microscopy of parasites

The ultramorphological identification of detected parasites was performed using SEM. The gills of necropsied fish were fixed with 2.5% glutaraldehyde as described by Di Azevedo et al. [26], and dehydrated in serial ascending ethanol concentrations of 50%, 70%, 90%, and 100% for 10 min in each concentration. Next, the gills were dried using a CO_2_ critical point dryer (Autosamdri-815, Germany). The gills were stacked onto the stubs of the SEM and coated with gold (20 nm) in a sputter coater (Spi-Module sputter coater, UK). The processed gill specimens were photographed using a JSM 5200 electron probe microanalyzer (JEOL, Japan) at the Faculty of Agriculture, Cairo University, Egypt [23]. The measurements are shown in millimetres.

### 2.4. Bacterial isolation and identification

Seabass frys (n = 100) were anesthetized with 0.2 mg/mL (0.2% MS-222; Sigma). Frys were disinfected externally by immersion in 0.1% (w/v) benzalkonium chloride prepared in 1.5% (w/v) saline for 10s and washed three times in 0.85% (w/v) saline. Every five frys were manually homogenized in 1 mL sterile saline in a glass potter blender, and 100 µL of the homogenates were plated on marine agar plates (Difco Laboratories, USA) and thiosulphate citrate bile salt sucrose (TCBS) agar (Oxoid™). The plates were incubated overnight at 30°C. Randomly selected colonies were pure-cultured and phenotypically identified using Gram staining, growth on TCBS, sensitivity to O/129 (2,4-diamino-6,7-di-iso-propylpteridine phosphate) disk, and API 20E identification system. Isolates were stored in tryptone soy broth with glycerol at −80°C in a freezer.

### 2.5. *Molecular identification of* A. ocellatum

The protozoans were collected from the skin and gills of diseased fish. The collected protozoans were washed with distilled water several times to eliminate mucus or debris and centrifuged at 2,000 × *g* for 15 min. The washed protozoans were preserved at −20°C in Eppendorf tubes for further genetic analysis. The extraction of parasitic DNA was performed using the QIAamp DNeasy blood and tissue kit (Qiagen, Hilden, Germany). The extracted DNA was well-maintained in sterilized Eppendorf tubes at −20°C for PCR and sequencing purposes.

The amplification of (ITS) ribosomal DNA (rDNA) regions of Amyloodinium protozoan was performed using the following primer pair: ITSR: 5′-TCCCTGTTCATTCGCCATTAC-3′ and Dino5′UF: 5′-CAACCTGGTGATCCTGCCAGT-3′, as designated by Levy et al. [27]. The ITS rDNA regions were flanked by 18S and 28S rDNA genes. The PCR amplification technique was achieved using 1× PCR mix consisting of 200 mM (dNTP), 1 U Taq polymerase, 250 ng genomic DNA, and 0.25 μM of both primers in a final volume of 25 μL. Briefly, PCR was achieved under the following circumstances: initial denaturation for 2 min at 94°C, followed by 45 cycles of (94°C for 30 s, 59°C for 50 s, and 72°C for 2 min), and a final extension phase for 10 min at 72°C. The PCR products were purified using the QIAquick PCR Purification Kit (Qiagen) to assist in gene sequencing. The purified amplicons were commercially sequenced by Sigma Company using ABI 3730XL DNA sequencer in both directions.

### 2.6. *Molecular examination of* V. alginolyticus

DNA was extracted from purified bacterial isolates using the QIAamp DNA Mini kit (Qiagen) according to the manufacturer’s protocol. The extracted DNA was kept at −20°C for further molecular assays. The internal fragments of the 16S rRNA gene were amplified using the universal primers (27 F: 5′-AGAGTTTGATCCTGGCTCAG-3′ and 1492 R: 5′-GGTTACCTTGTTACGACTT-3′) described by Moreno et al. [28]. The PCR amplification technique was achieved using 1X PCR mix consisting of 200 mM dNTP, 1 U Taq polymerase, 250 ng genomic DNA, and 0.25 μM of both primers in a final volume of 25 ul. The cycling conditions were adjusted at the following settings: initial denaturation at 94°C for 35 s, 55°C for 50 s, and 72°C for 1 min, and a final extension phase at 72°C for 10 min. The PCR products were purified using the QIAquick PCR Purification Kit to assist in gene sequencing. The purified amplicons were commercially sequenced by Sigma using an ABI 3730XL DNA sequencer in both directions.

### 2.7. Phylogenetic analysis

The raw sequences of the *A. ocellatum* and *V. alginolyticus* were edited and assembled using the BioEdit program v7.2.5 [29]. The assembled sequences were compared to the other sequences available in GenBank through BLASTN. The sequences of the ITS rDNA regions of Amyloodinium protozoans and a fragment of the 16S rRNA gene of *V. alginolyticus* were deposited in GenBank. To calculate genetic distance, the phylogenetic trees were performed using MEGA X with the neighbour-joining method of the Kimura two-parameter model, and the level of confidence was tested by bootstrap analysis for each branch at 1,000 repeats [30].

### 2.8. Quantitative Real-time Polymerase Chain Reaction (qRT-PCR)

#### 2.8.1. Sampling

The infected gill samples (0.5 cm^2^) were aseptically dissected. Samples from five uninfected fish were collected and used as negative controls; samples were classified as infected gills with *V. alginolyticus* and *A. ocellatum* and infected gills only with *V. alginolyticus.*

#### 2.8.2. RNA isolation

The mRNA from 100 mg infected gills was isolated using the total RNA isolation kit (Ambion; Applied Biosystems) according to the manufacturer’s instructions. Homogenization of the gills was applied using Lysing Matrix D tubes (MP Biomedicals) and a FastPrep-24 homogenizer (MP Biomedicals; two cycles of 30 s at 6 m/s). The purity and quantity of mRNA were measured using Nanodrop (Thermo Scientific). A 500 ng mRNA was handled with DNase I amplification grade (Invitrogen) according to the manufacturer’s instructions. The purified mRNA was reverse transcribed by the High-Capacity cDNA Archive Kit (Applied Biosystems) according to the manufacturer’s protocol [31,32].

#### 2.8.3. Quantitative real-time PCR protocol (qRT-PCR)

The PCR primer sets specific for tumour necrosis factor-α (TNF-α), interleukin (IL)-1β, and IL-6 specific for *D. labrax* were prepared as deposited in GenBank (Table 1). β-Actin was used as a reference gene and subsequently used for sample normalization [33,34]. A Step One™ Real-time PCR System (Applied Biosystems, USA) was used to perform quantitative PCR tests. In the reaction, 10 µL SYBR® Premix Ex Taq™ (Tli RNase H Plus), ROX plus (TaKaRa, Japan), 1 µL cDNA, and 0.5 μL of forward and reverse primers (100 nM) were combined and filled to a final volume of 20 μL with ultrapure water [35]. The RT-PCR methodology was run as follows: denaturation for 30 s at 94°C, annealing for 30 s at 60°C, and extension for 45 s at 72°C, in a 40-cycle amplification. The ∆CT value was calculated by the subtraction of β-actin CT as an internal control, in which CT is the cycle number at which detectable signals are achieved [36].

**Table 1.** Primer sequences of gene used for gene expression analysis

Gene	Primer sequence (5’ – 3’)	Accession number
**IL-1β**	F-ATCTGGAGGTGGTGGACAAA	AJ311925
	R-AGGGTGCTGATGTTCAAACC	
**TNF-α**	F-AGCCACAGGATCTGGAGCTA	DQ200910
	R-GTCCGCTTCTGTAGCTGTCC	
**IL-6**	F- ACTTCCAAAACATGCCCTGA	AM490062
	R-CCGCTGGTCAGTCTAAGGAG	

### 2.9. Antibiogram

Bacterial isolates were cultured in tryptic soy broth supplemented with 2% NaCl for 24 h at 30°C. The bacterial culture was diluted to an absorbance of 0.6 at 610 nm with phosphate-buffered saline (pH 7.2) and 0.1 mL spread onto Mueller–Hinton agar. The plates were left to dry, and the following antibiotic disks were placed onto the streaked plates and incubated for 24 h at 30°C: ampicillin 10 µg, amoxycillin 30 μg, gentamycin 10 μg, trimethoprim/sulfamethoxazole 1.25/23.75 μg, florfenicol 30 µg, ciprofloxacin 5 μg, and tetracycline 30 μg (Oxoid™). The bacterial growth inhibition zones were scored, and the results were interpreted as susceptible (S), intermediate (I), or resistant (R) according to the method described by Bauer et al. [37] and CLSI [38].

### 2.10. Field treatment trial

An improved therapeutic regimen using a combination of treatments [florfenicol, hydrogen peroxide (H_2_O_2_), and copper sulphate] was applied to control infections affecting European seabass frys. According to Wang et al. [39], florfenicol at (20 mg/kg body weight) was mixed with the feed (Aller, Egypt) using fish oil and offered to seabass frys for 10 successive days. According to Montgomery-Brock et al. [40], H_2_O_2_ was added to tank water at 25 mg/L for 30 min/day for 3 successive days. According to Francis-Floyd and Floyd [41], copper sulphate was used as a prolonged bath at 0.2 mg/L for the next 7 days. Therapeutic efficacy was determined by monitoring fish mortalities and parasitological and bacteriological examinations of random fish samples at the end of treatments.

### 2.11. Histopathological examination

Tissue specimens were collected, fixed in 10% neutral buffered formalin, washed, dehydrated, cleared, and embedded in paraffin. The paraffin-embedded blocks were sectioned at 5 μm thickness and stained with haematoxylin and eosin, Giemsa stain, and modified Gram stain [42] for histopathological examination. The stained sections were examined by a light microscope (BX50; Olympus, Japan).

### 2.12. Statistical analysis

The results of gene expression were analysed using one-way-ANOVA test. The data were considered statistically significant when *p* < 0.05. Statistical analysis was done using SPSS software (SPSS, Inc., Chicago, IL, USA).

### 2.13. Ethics approval

This study was approved by the Institutional Animal Care and Use Committee of Cairo University (ethical approval no. CU II F 28 21).

## 3. Results

### 3.1. Clinical examination

Eighty-five of 100 frys were clinically diseased. Clinically diseased *D. labrax* frys demonstrated lethargy, dark discoloration, and sluggish swimming behaviour. Velvety skin appearance was commonly noticed. Fish showed signs of respiratory distress, including piping behaviour and rapid opercular movement. Abdominal swelling and ascites were also noticed. Some fish died without showing any apparent gross lesions.

### 3.2. Parasitological examination

#### 3.2.1. *Morphological identification and electron microscopy of* A. ocellatum

**Figure 1.**
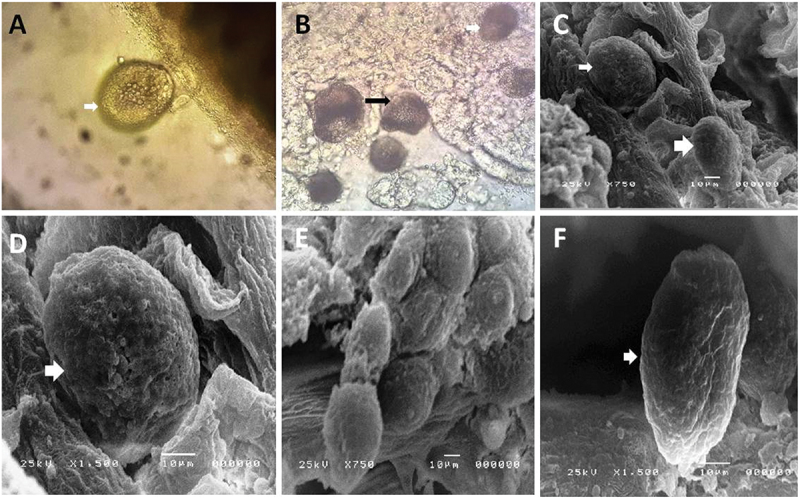
**A, B**: fresh smears of gills showing *A. ocellatum* trophont which appear as dark brown with pigment inside it. **C-F**: Scanning electron micrograph of *A. ocellatum* trophont which was spherical; oval; or elliptical to pear in shape which appear attached firmly on the gills filaments and present in clusters. **F**: the trophont appear pear shape with stalk to attach firmly on the gills.

The detected trophonts of *A. ocellatum* were spherical, oval, or elliptical to pear in shape and attached firmly to the gills or the skin surface. Its length ranged from 50 to 135 (90.69 ± 2.58). The cytoplasm was opaque brown and appeared as a sac filled with granules. The protozoan presents with rhizoids or stalks through which it attaches firmly to the host tissue (Figure 1).

#### 3.2.2. *Molecular identification of* A. ocellatum

**Figure 2.**
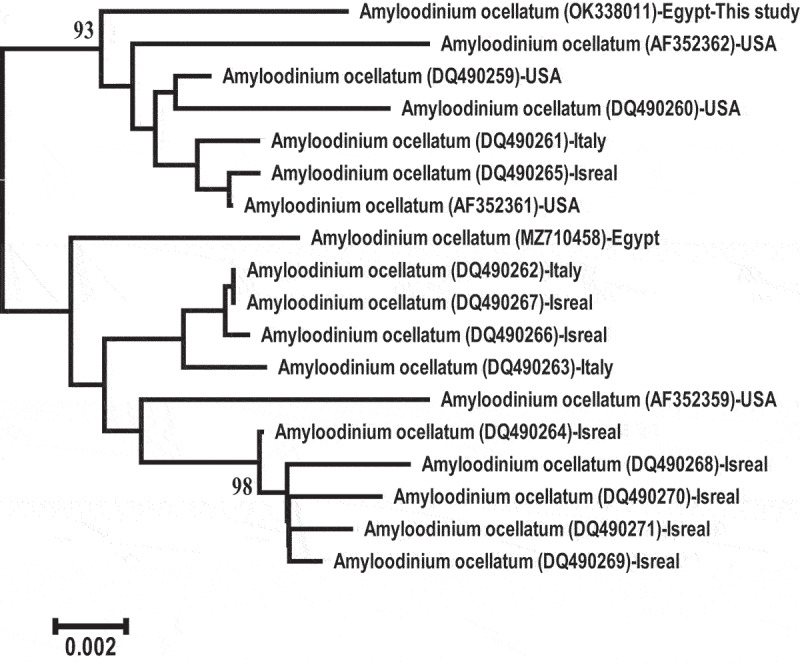
The phylogenetic tree of ITS rDNA regions displayed the comparative analysis sequence of *A. ocellatum* infecting *D. labrax.*

The ITS rDNA sequencing of this parasite yielded 1,322 bp and was deposited in GenBank (accession no. OK338011). Depending on the analysis of the present sequence, this parasite is firmly embedded within the family Oodiniaceae and identified as *A. ocellatum*. Accession number OK338011 showed 98.71% to 98.26% similarity with *A. ocellatum* (AF352361.1, DQ490265.1, DQ490261.1, AF080096.1, AF352360.1, DQ490260.1, and MZ710458.1). The phylogenetic tree of the ITS regions of this *A. ocellatum* revealed two main clusters (Figure 2). The first cluster showed that *A. ocellatum* sequence retrieved in this study was grouped with *A. ocellatum* sequences from Italy, United States and Israel.

### 3.3. *Prevalence of* A. ocellatum *infections*

Most of the examined *D. labrax* specimens (85%) were infected with *A. ocellatum*. The trophont of *A. ocellatum* was detected in the gills and skin of infected *D. labrax*. The different parasitic intensities were recorded in infected fish, ranging from mild (<5), moderate [5–10], and severe [10–20] trophonts per microscopic field (Table 2). No. of other ectoparasitic infestations were noticed in the investigated specimens.

**Table 2.** Prevalence, intensity and pattern of *A. ocellatum* in *D. labrax.*

	No. examined fish	No. infected fish	Co-infection with *A. ocellatum* and *V. alginolyticus*		Patterns of parasitic Intensity		
					Skin	Gills	%
			Mixed *A. ocellatum* + *V. alginolyticus*					Single *A. ocellatum*
*A. ocellatum*	100	85	75	10	+++	++	25
					++	+	15
					++	++	35
					+++	+++	10

Prevalence % was calculated to according to the total number of examined fish. Examined fish were recorded as positive when 1 parasitic trophont was detected. The degree of protozoan intensity was categorized according to number of trophonts per examination field as the following: + (<5); ++ [5–10]; +++ [10–20]

### 3.4. Bacteriological examination

#### 3.4.1. *Prevalence of* V. alginolyticus

*V. alginolyticus* was detected in all tested specimens (pooled samples), except for three groups that were negative (15 fish). Fifteen *V. alginolyticus* isolates were obtained from the investigated specimens. All fish infected with *V. alginolyticus* were coinfected with *A. ocellatum* (75%), whereas only 10% was infected only with *A. ocellatum*. Isolates were Gram-negative, bacilli, oxidase-positive, and sensitive to O/129 vibriostatic disc. The isolates produced yellow colonies on TCBS agar.

### 3.5. *Molecular identification of* V. alginolyticus

**Figure 3.**
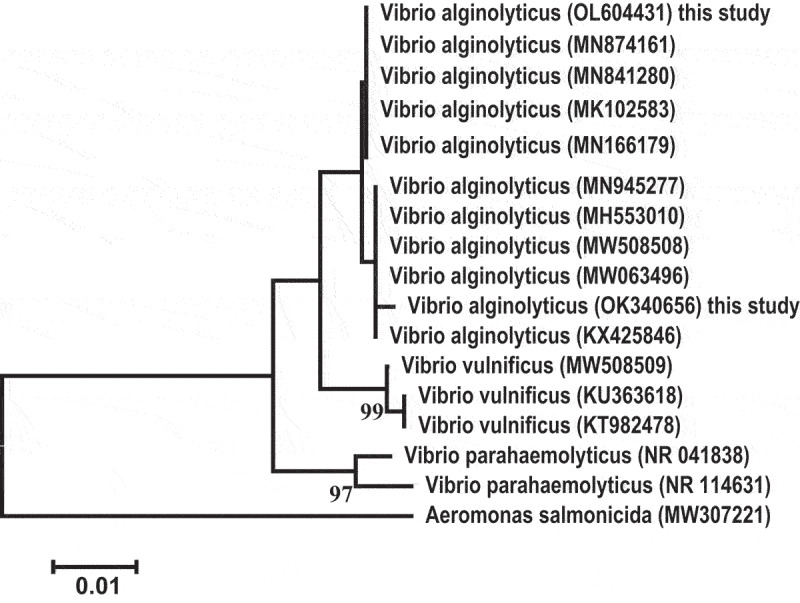
The phylogenetic tree exhibited the comparative analysis of 16S rRNA sequence of *V. alginolyticus* infecting *D. labrax.*

The assembled 16S rRNA gene sequence of two *V. alginolyticus* strains was submitted to GenBank (accession nos. OK340656 and OL604431). Depending on sequence analysis, the current sequences were confirmed to be *V. alginolyticus. V. alginolyticus* (OK340656 and OL604431) from diseased fish samples exhibited 100% to 99.93% identity with *V. alginolyticus* (MN874161.1-MN841280.1-MK102583.1-MN166179.1-MN945277.1). The neighbour-joining phylogenetic tree constructed based on the sequenced 16S rRNA genes of *V. alginolyticus* was grouped with their relevant *V. alginolyticus* sequences which were genetically apart from other related species (*V. vulnificus* (99%) and *V. parahaemolyticus* (97%) depending on their degree of similarities (Figure 3).

### 3.6. TNF-α, IL-1β, and IL-6 gene expression

**Figure 4.**
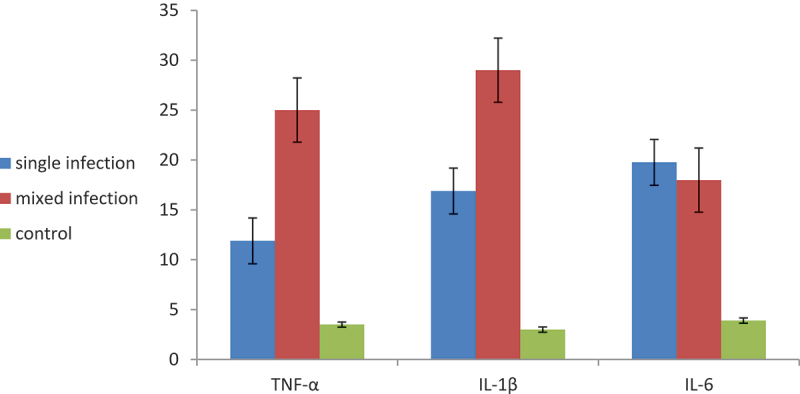
Expression of tumour necrosis factor alpha (TNF-α) and interleukin 1; interleukin- 6, genes in *D. labrax* gills singly infected with *A. ocellatum* or co-infected with *A. ocellatum* + *V. alginolyticus.*

TNF-α mean was 25.00 ± 0.98 in fish coinfected with *A. ocellatum* and *V. alginolyticus* and 11.89 ± 0.64 in fish gills infected only with *A. ocellatum*. IL-1β was 29.00 ± 1.58 in fish coinfected with *A. ocellatum* and *V. alginolyticus* and 16.88 ± 0.75 in a single infection with *A. ocellatum*. IL-6 was 19.76 ± 1.58 in the mixed *A. ocellatum* and *V. alginolyticus* infection and 17.98 ± 0.75 in fish gills infected only with *A. ocellatum* (Figure 4).

### 3.7. Antibiogram

Isolates expressed different sensitivity patterns against tested antibiotics. All isolates were resistant to ampicillin 10 μg, amoxycillin 30 μg, and gentamycin 10 μg. Florfenicol 30 µg was the most effective antibiotic against *V. alginolyticus* isolates, followed by ciprofloxacin 5 µg and trimethoprim/sulfamethoxazole 1.25/23.75 μg (Table 3).

**Table 3.** Antibiotic sensitivity testing of *V. alginolyticus* isolates

Item	S	I	R
Ampicillin 10 µg	-	-	20
Amoxycillin 30 µg	-	-	20
Gentamycin 10 µg	-	-	20
Trimethoprim 1.25 μg / Sulfamethoxazole 23.75 μg	7	12	1
Ciprofloxacin 5 µg	11	8	1
Florfenicol 30 µg	14	6	-
Tetracycline 30 µg	6	10	4

(S) Sensitive; (I) intermediate sensitive; (R) resistant; (-) no isolates

### 3.8. Treatment trial of the diseased seabass frys

Application of the florfenicol, H_2_O_2_, and copper sulphate therapeutic regimen reduced *D. labrax* mortalities. The health and survival of treated *D. labrax* frys were enhanced, as shown by normal swimming behaviour and improvement of feed intake. Mortalities dropped to 10% after treatments compared to 70% before chemotherapeutic application. The *A. ocellatum* trophont count in smears prepared from fish gills and skin collected from random samples decreased considerably and disappeared at the end of treatments. Parasitological and bacteriological examinations of random fish samples collected from tanks after the 10-day treatment period showed the absence of both trophonts and *V. alginolyticus*, indicating the effectiveness of therapy.

### 3.9. Histopathological findings

**Figure 5.**
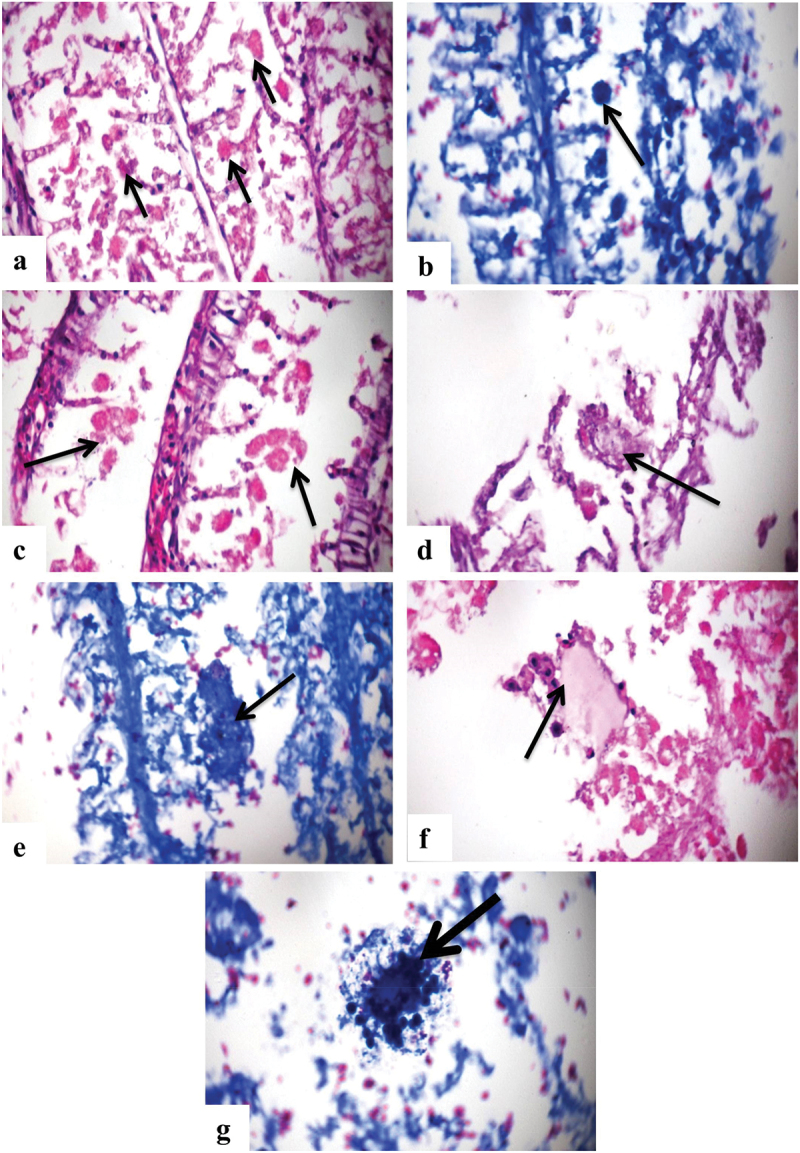
Photomicrograph of gills showing: a) showing different developmental stages of *A. ocellatum* attached to secondary gill lamellae (arrows) (H&EX200). b) Giemsa stained sections illustrating the parasitic infestation of the gills (arrow) (Giemsa X200). c) higher magnification of a) showing the tomont stage (4 cells) of the parasite (arrows) (H&EX400). d) higher magnification of a) illustrating another tomont stage (more than 4 cells) (arrow)(H&EX400). e) Giemsa stained sections showing the tomont stage of the parasite (arrow) (Giemsa X400). f) The trophont stage of the parasite (arrow) (H&EX1000). g) Giemsa stained sections to confirm the presence of trophont stage of the parasite attached to secondary gill lamellae (arrow)(Giemsa X1000).

Histopathological examination of tissue sections revealed large number of oval to spherical bodies representing the different developmental stages of *A. ocellatum* attached to secondary gill lamellae (Figure 5a). Protozoal infection of gills was confirmed by Giemsa-stained tissue sections (Figure 5b & e). Some sections revealed the presence of different tomont stages of the protozoan (4 and 16 cells) (Figure 5c & d). Other sections showed the structure of the trophont stage of the parasite attached to secondary lamellae with multiple vacuoles (Figure 5f), also confirmed by tissue Giemsa stain (Figure 5g). The protozoan infected the gills with no tissue reaction of the gills.

**Figure 6.**
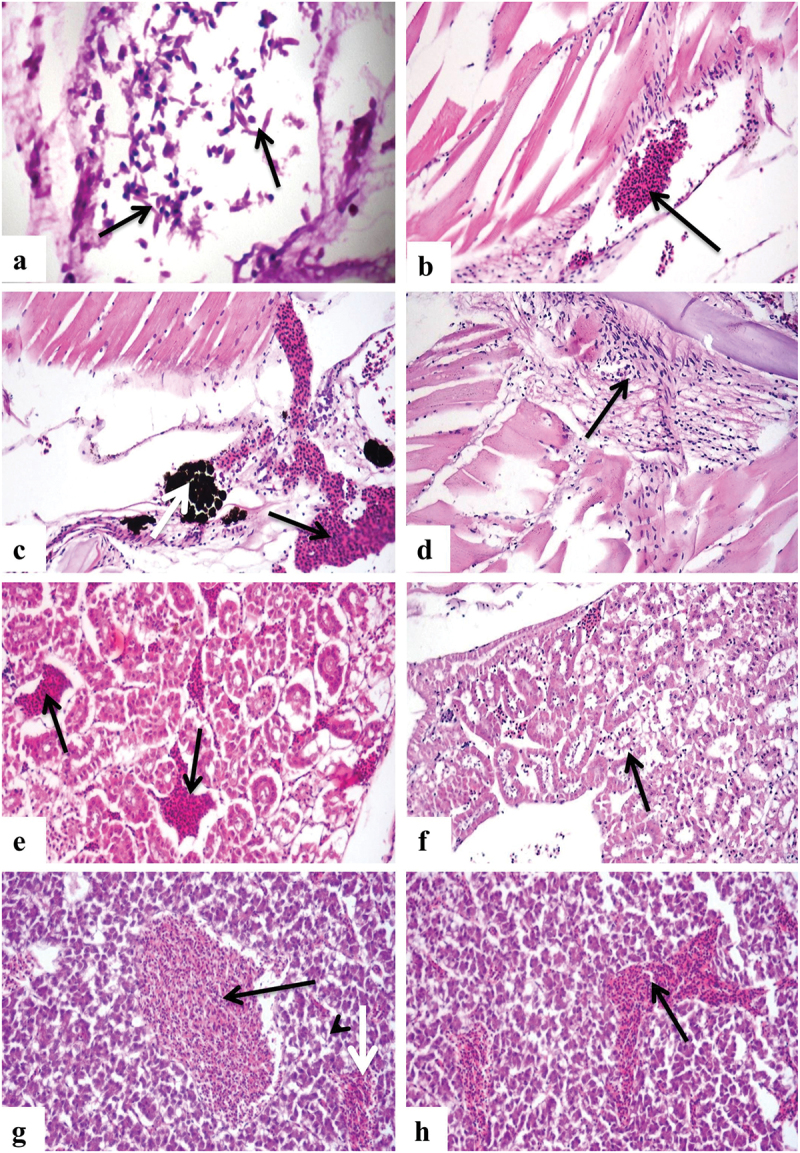
Photomicrograph showing: a) Gram staining of tissue showing presence of Gram negative slightly curved rods of *Vibrio* spp. in-between RBC’s (arrows) (Gram stainX1000). b) European seabass muscles showing congestion of interstitial blood vessel (arrow) (H&EX400). c) European seabass Muscles exhibiting haemorrhages (black arrow) and proliferation of melanomacrophages (white arrow) (H&EX400).d) muscles showing interstitial oedema with infiltration of mononuclear inflammatory cells (arrow) (H&EX400). e) Renal tissues showing congestion of interstitial blood vessels (arrows) (H&EX400). f) Kidney showing vacuolation and necrosis of tubular lining epithelium (arrow) (H&EX400). g) Hepatic tissues showing congestion of central vein (black arrow) and sinusoids (white arrow) and vacuolation of hepatocytes (arrowhead) (H&EX400). h) Liver showing haemorrhage in-between hepatocytes (arrow) (H&EX400).

Modified Gram staining of the tissue revealed Gram-negative slightly curved rods (Figure 6a). Histopathological examination of muscles and different internal organs revealed multiple histopathological alterations related to Vibrio infection. The most common lesions were severe haemorrhage, congestion, degeneration, and necrosis of parenchymatous organs. Muscles showed congestion of interstitial blood vessels (Figure 6b) with severe haemorrhage and proliferation of melanomacrophages (Figure 6c), and interstitial tissue showed oedema with infiltration of mononuclear inflammatory cells (Figure 6d). Renal tissue exhibited interstitial blood vessel congestion (Figure 6e). The lining epithelium of renal tubules showed vacuolation and necrosis, most with few mononuclear inflammatory cell infiltrations (Figure 6f). Histopathological examination of hepatic tissue showed severe congestion of the central vein and blood sinusoids. Some hepatocytes showed vacuolar degeneration (Figure 6g), and some liver sections showed extensive haemorrhage replacing hepatocytes (Figure 6h).

## 4. Discussion

Fish mass mortalities due to infectious agents represent a puzzling dilemma in mariculture industry worldwide. In this study, parasitological and bacteriological studies have suggested that both *V. alginolyticus* and *A. ocellatum* were the pathogenic agents incriminated in the high seabass frys mortality. These infectious agents are among the most dangerous infections targeting the European seabass aquaculture [9,19,20,43,44].

In this study, dissolved oxygen and unionized ammonia values recorded during the eruption of the clinical disease were unfavourable. The vulnerability of fish to infections increased during their exposure to stressful circumstances, such as high stocking densities, inferior water quality, or abrupt changes in water quality measures [45,46]. Therefore, the unfavourable water quality parameters recorded during the mortalities were believed to have predisposed European seabass frys to reported pathogenic agents that worked in synergy to induce the recorded higher mortalities [10,46].

*V. alginolyticus* was detected in all tested specimens (pooled samples; 5 in each group), except for the three negative groups. Similarly, Moustafa et al. [9] recorded a high *V. alginolyticus* prevalence in rigorous mortality outbreaks affecting maricultured *D. labrax* in earthen ponds and floating net cages in Northern Egypt. This bacterial agent is a critical pathogen for numerous fish species, including seabass and seabream in the Mediterranean either in natural fisheries or captivity, causing colossal mortality [9,12].

External protozoal infections, such as *A. ocellatum*, can produce wounds and injuries on the external surfaces of fish, which are believed to be effective portals of entry for *Vibrio* spp [47]. In this study, all fish infected with *V. alginolyticus* were coinfected with *A. ocellatum*, suggesting a role for such protozoal infections in the emergence of vibriosis affecting *D. labrax*. Previous studies showed a direct link between bacterial and parasitic infections [10,48]. Heavy infections with *A. ocellatum* can adversely affect the health status and feeding behaviour of fish, with consequent death due to acute anoxia and asphyxia [49]. Attachments, inflammation, haemorrhages, necrosis, and other lesions induced by *A. ocellatum* can potentiate numerous opportunistic bacterial infections like *V. alginolyticus*. Parasites often gain entry to aquaculture facilities due to insufficient quarantine and biosecurity measures. Infected broodstocks, contaminated water, and food are potential sources of infections [50,51]. *A. ocellatum* can be transmitted through direct contact with live dinospores and via aerosolized droplets [16].

Vibrio infection starts with bacterial adhesion to fish mucus, proliferation, and colonization of epithelial tissues. Vibrios secrete numerous toxic hydrolytic enzymes, potentiating further pathogenic invasions into tissues and the initiation of septicaemic infections [17]. The greater part of *D. labrax* specimens (70%) was infected with *A. ocellatum*. The protozoan was identified in fish skin and gills, and the parasite intensity ranged between 5 and 20/microscopic fields). Frys kept at high densities in tanks are commonly confined to a small area providing favourable circumstances for parasitic infections [16].

In this study, the identity of *V. alginolyticus* was genetically identified by sequencing the 16S rRNA gene. The 16S rRNA gene sequence of *V. alginolyticus* was virtually (>99%) identical to its correlated bacteria. The genetic characterization of the current protozoan took place by sequencing the ribosomal internal transcribed spacer regions (ITS rDNA) for *A. ocellatum*. The ITS rDNA regions flanking the small subunit (18S rDNA) and large subunit rDNA genes (28S rDNA) are significant genetic markers for recognizing fish protozoans. Several studies successfully used these genes to genetically identify *A. ocellatum* [27].

To express the immune genes, the three different genes, IL-1β, IL-6, and TNF-α, were up-regulated during infection with *A. ocellatum* due to the immune reaction of the fish body against infection with either protozoal parasites alone or coinfection with *V. alginolyticus*. Fish acquire immunity by identifying different foreign bodies, such as bacteria, viruses, and parasites, with the aid of different immune cells, such as macrophages, eosinophils, and lymphocytes (T or B cells) [52]. The three genes were elevated during infection with *A. ocellatum* for presenting the antigen to T cells to complete the immunological function and produce different cytokines. The results were consistent with Nozzi et al. [43], who discovered an increase in interleukin and TNF-α in *A. ocellatum*-infected gills.

Frequent and irresponsible applications of antibiotics in fish farming to control infections resulted in the emergence of uncountable resistant bacterial strains. The retrieved isolates exhibited different patterns of sensitivity against tested antibiotics. According to antibiotic sensitivity testing, florfenicol 30 µg was the most effective antibiotic against *V. alginolyticus* isolates; therefore, it was used as the core systemic therapy with H_2_O_2_ and copper sulphate to control infections in the studied hatchery. Previous research showed that florfenicol is effective against vibriosis *in vivo* [52,53]. Copper sulphate is the drug of choice for controlling *A. ocellatum* in aquaculture [54]. However, some previous studies showed that copper sulphate alone is not enough to control amyloodiniosis [55]. Therefore, H_2_O_2_ was used with copper sulphate to competently control *A. ocellatum* infestations in this study. According to Montgomery-Brock [40], H_2_O_2_ is highly efficient against *A. ocellatum*. The therapeutic regime (florfenicol, H_2_O_2_, copper sulphate) controlled infections and reduced fish mortality from 70% to 10% after receiving the prescribed chemotherapeutics.

In this study, histopathological alterations suggest mixed bacterial and parasitic infections. Sections prepared from gills revealed many different developmental stages of *A. ocellatum* attached to secondary gill lamellae [43,44]. Gram-stained tissue sections revealed the presence of Gram-negative, slightly curved rods suggestive of *Vibrio* infection. Histopathological lesions exhibited through internal organs are frequent in bacterial septicaemic infections. Congestion, haemorrhage, degeneration, and necrosis were observed in tissue, correlating with previous findings in Asian seabass, *Lates calcarifer* [56]. Tissue damage is relevant to the release of bacterial extracellular enzymes, such as protease, which demonstrate a critical role in the pathogenesis of vibriosis [17,56,57].

Economic losses associated with marine fry mortalities are very costly when fry rearing costs (feeding, running, management, and veterinary care) are considered. Thus, investigating the triggers and real aetiologies behind such mass mortalities is highly mandated to decisively propose the best corrective action. There are too many marine hatcheries relying on open seawater in Egyptian coastal provinces are found, and these hatcheries are very vulnerable to pathogenic biological invasions originating from natural open water. *A. ocellatum* is a very common protozoal parasite that extensively hammers marine fish frys collected from the wild or reared on natural seawater. It is known to swiftly damage the skin’s immune barriers, with consequent pathogenic bacterial invasions, *V. alginolyticus* ubiquitously exists in the shallow seawater supply of the Egyptian coastal marine basin, complicating the above parasitic invasion with the ultimate result of heavy mass kills among vulnerable hatchery-reared frys. Ultimately, wise and/or swift therapeutic intervention is vital in restoring hatchery resources and securing a successful rearing cycle.

## 5. Conclusion

*A. ocellatum* is a serious parasitic invader that drastically suppresses the primary physical barriers, which triggers vigorous attacks of the ubiquitous pathogenic *V. alginolyticus*. Erratic managerial practices and unfavourable water quality parameters drive fish frys to be highly susceptible to various pathogenic invasions, including the nasty *V. alginolyticus*. Erratic/irresponsible applications of antibiotics in aquaculture together with random dumping off municipal sewage into water streams will possibly result in the emergence of resistant bacterial strains among fishes reared in facilities irrigated by such streams. The adjusted application of water dependent disinfectants as well as an oral therapeutic regimen utilizing FDA-approved oral antibiotics such as florfenicol was very effective in controlling the disease outbreak. Ultimately, adopting strict biosecurity measures pertaining to biological filtering and UV treatment of seawater before passage into hatchery rearing tanks will sharply minimize these pathogenic invasions.

## Data Availability

All data generated or analyzed during this study are included in this published article
